# Linker-Free Hyaluronic Acid-Dexamethasone Conjugates: pH-Responsive Nanocarriers for Targeted Anti-Inflammatory Therapy

**DOI:** 10.3390/ijms26146608

**Published:** 2025-07-10

**Authors:** Anton N. Bokatyi, Natallia V. Dubashynskaya, Igor V. Kudryavtsev, Andrey S. Trulioff, Artem A. Rubinstein, Elena N. Vlasova, Yury A. Skorik

**Affiliations:** 1 Branch of Petersburg Nuclear Physics Institute Named by B.P. Konstantinov of National Research Centre “Kurchatov Institute”—Institute of Macromolecular Compounds, Bolshoi VO 31, Saint Petersburg 199004, Russia; 2 Institute of Experimental Medicine, Acad. Pavlov St. 12, Saint Petersburg 197376, Russia

**Keywords:** hyaluronic acid, dexamethasone, polymeric drug delivery systems, anti-inflammatory activity

## Abstract

The covalent conjugation of pharmaceutical compounds to polymeric carriers represents an effective strategy for enhancing drug properties, including improved bioavailability, targeted delivery, and sustained release, while reducing systemic toxicity and adverse effects. By exploiting the physicochemical characteristics of biopolymers—particularly molecular charge and weight—we engineered a polymeric platform for glucocorticoid delivery with precisely controlled parameters including particle size, surface charge, targeting capability, and release kinetics. This study reports a linker-free synthesis of hyaluronic acid-dexamethasone (HA-DEX) conjugates through Steglich esterification, catalyzed by 4-dimethylaminopyridine (DMAP), which facilitates the acylation of sterically hindered alcohols. The reaction specifically couples carboxyl groups of hyaluronic acid with the C21 hydroxyl group of dexamethasone. Incorporation of hydrophobic dexamethasone moieties induced self-assembly into nanoparticles featuring a hydrophobic core and negatively charged hydrophilic shell (−20 to −25 mV ζ-potential). In vitro characterization revealed pH-dependent release profiles, with 80–90% dexamethasone liberated in mildly acidic phosphate buffer (pH 5.2) versus 50–60% in phosphate-buffered saline (pH 7.4) over 35 days, demonstrating both sustained release and inflammation-responsive behavior. The conjugates exhibited potent anti-inflammatory activity in a human tumor necrosis factor-α (TNFα)-induced inflammation model. These findings position HA-DEX conjugates as promising candidates for targeted glucocorticoid delivery to specific anatomical sites including ocular, articular, and tympanic tissues, where their combination of CD44-targeting capability, enhanced permeability and retention effects, and stimulus-responsive release can optimize therapeutic outcomes while minimizing off-target effects.

## 1. Introduction

Glucocorticoids, including dexamethasone (DEX), prednisolone, and hydrocortisone, rank among the most prescribed steroidal anti-inflammatory agents for treating diverse inflammatory pathologies [1,2]. However, these agents induce significant adverse effects—particularly during long-term systemic administration—and exhibit low bioavailability in isolated organs (e.g., joints, eyes, and ears) following oral or parenteral delivery [3,4]. To enhance local bioavailability and reduce systemic toxicity, site-specific injections (e.g., intra-articular, intraocular, or intratympanic) are employed. Yet these procedures entail technical complexity and risk complications such as infectious lesions or retinal detachment if improperly executed [5,6,7]. Consequently, developing polymeric glucocorticoid delivery systems based on safe, biodegradable biopolymers for sustained drug release is critically important. Incorporating glucocorticoids into biopolymer matrices enables programmable drug release kinetics, prolongs therapeutic residence at target sites, and reduces dosing frequency-related side effects [8,9,10]. Systems providing release profiles spanning days to months are optimal [11]; chemically grafted conjugates represent promising nanocarriers, as the hydrolytic cleavage of covalent bonds facilitates delayed release [12,13,14].

**Figure 1:**
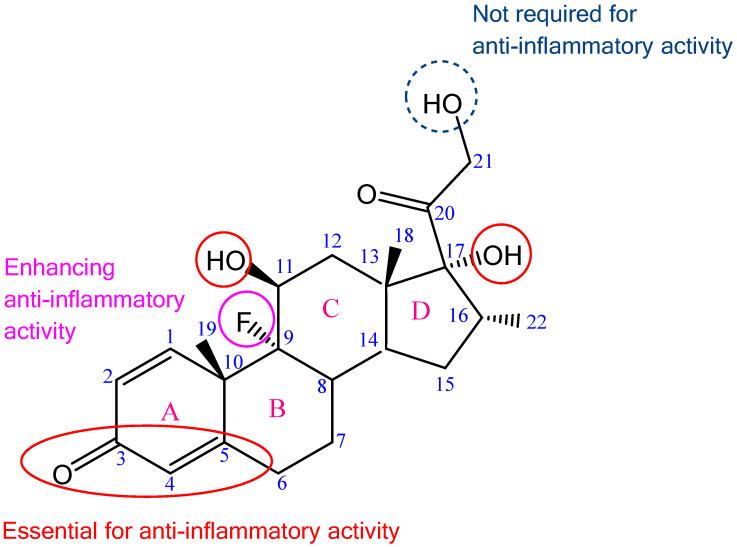
Chemical formula of DEX with highlighted structures responsible for glucocorticoid anti-inflammatory effect; adapted from [15,17].

DEX (Figure 1), a potent lipophilic glucocorticoid, is widely utilized for its anti-inflammatory, anti-rheumatic, and immunosuppressive efficacy [11]. As a fluorinated hydrocortisone homolog, its C9 fluorine atom confers ~30-fold greater glucocorticoid potency than cortisone while minimizing mineralocorticoid activity [15]. Key structural motifs—C3, C4–C5, C11, and C17—underpin its anti-inflammatory action. The double bond between carbon atoms 1 and 2 of the steroid core and the C16 methyl group enhance glucocorticoid activity while reducing Na^+^ retention, and the C9 fluorine impedes metabolic degradation [15,16,17]. The C21 hydroxyl group, nonessential for activity, is frequently exploited for prodrug design (e.g., DEX sodium phosphate) and biopolymer conjugation [16,18,19,20]. DEX treats acute/chronic conditions (e.g., arthritis, edema, ocular allergies, cochlear damage, etc.) via intravenous, oral, or topical routes [15,21]. Natural polysaccharide-based delivery systems for DEX can prolong release, enhance local retention, and minimize systemic exposure [22,23,24]. Conjugation also elevates molecular weight, enabling passive targeting to inflammation sites via the enhanced permeability and retention (EPR) effect [25].

Synthetic strategies for DEX–polysaccharide conjugates include carbodiimide activation, RAFT polymerization, and click chemistry [14]. These facilitate grafting via hydrolyzable amide/ester linkages. For instance, anhydrides (succinic, glutaric) introduce carboxyl groups onto DEX for amide bonding with chitosan [26]. Alternatively, Steglich esterification using dicyclohexylcarbodiimide/4-dimethylaminopyridine (DMAP) enables the direct conjugation of DEX’s C21 hydroxyl to carboxylated polysaccharides (e.g., HA), forming pH-sensitive ester bonds without linkers [27].

Our group previously engineered succinyl-DEX–chitosan conjugates with succinyl modifications (64–68% succinylation; 2–4% DEX substitution) to impart anionic charge (ζ-potential of −30 to −33 mV) for parenteral/intravitreal delivery [13]. These nanoparticles (400–1100 nm) exhibited sustained release (8–10% cumulative DEX/succinyl-DEX over 1 month at pH 7.4) and suppressed CD54 expression in THP-1 cells by 2–4-fold in human tumor necrosis factor-α (TNFα)/lipopolysaccharide models.

Here, HA is selected as the polymeric carrier due to its exceptional biodegradability, biocompatibility [28], and physiological ubiquity (e.g., extracellular matrix, articular cartilage, synovial fluid, and ocular tissues) [29,30]. Anionic surface charge minimizes phagocytic clearance and enhances mobility in biological fluids—unlike cationic systems, which aggregate with anionic biomacromolecules in blood/synovial fluid [31]. HA’s polyanionic nature, controllable degradation [30], and CD44 receptor affinity (overexpressed at inflammation sites and on immune cells) [32] enable active targeting. HA–corticosteroid conjugates also self-assemble into nanoparticles that exploit passive EPR-mediated accumulation in inflamed/tumoral tissues [33,34,35,36]. For example, Chen et al. [37] synthesized HA–DEX conjugates (9.8 wt% DEX) via Boc-glycine linker chemistry; intravenous administration in lipopolysaccharide-induced lung inflammation models demonstrated CD44-mediated macrophage targeting and superior efficacy versus free DEX.

Despite these advances, existing conjugation methods often involve multi-step syntheses, hydrophobic linkers that alter drug pharmacology, or non-biodegradable bonds that impede native drug release. A streamlined, linker-free strategy yielding hydrolysable ester bonds could significantly improve clinical translatability by ensuring the direct release of active DEX while taking advantage of HA’s targeting capabilities. We hypothesize that conjugating DEX to HA via a one-step carbodiimide-mediated esterification at the C21 position will generate pH-responsive nanoparticles capable of (i) passive EPR-driven targeting to inflammation sites, (ii) CD44-mediated active delivery to immune cells, and (iii) sustained release of native DEX via ester hydrolysis. This approach is motivated by the urgent need for precision therapies that maximize therapeutic efficacy in isolated organs (e.g., joints, eyes, ear, etc.) while eliminating systemic toxicity—particularly for chronic conditions requiring long-term glucocorticoid intervention. This study synthesizes HA–DEX conjugates via direct esterification (using water-soluble 1-ethyl-3-(3-dimethylaminopropyl)carbodiimide hydrochloride (EDC) and 4-dimethylaminopyridine (DMAP)) to develop a versatile delivery platform for targeted applications—including local (ocular, articular, tympanic) and systemic (lung, tumor) administration. Physicochemical characterization, in vitro release, and preliminary biological evaluation will assess translational potential.

## 2. Results and Discussion

### 2.1. Synthesis and Characterization of HA-DEX Conjugates

**Figure 2:**
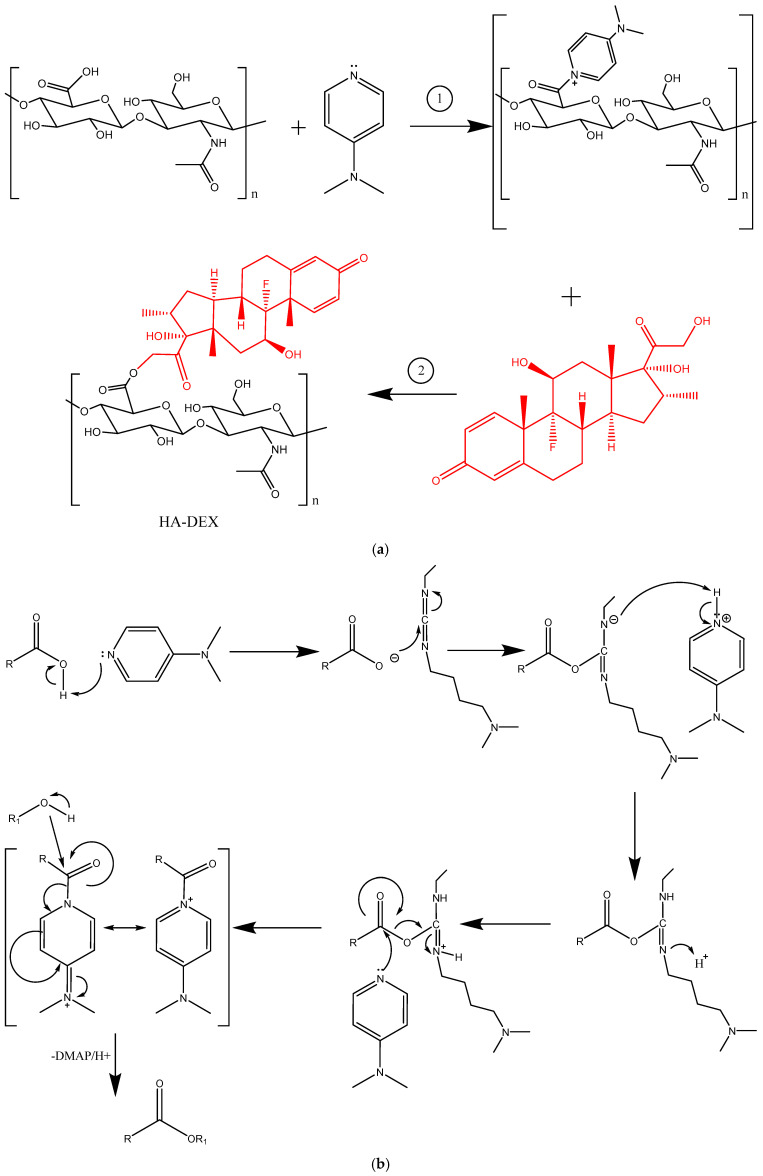
The synthesis scheme for HA-DEX (**a**) and the mechanism of esterification according to Steglich (**b**).

The development of sustained-release drug delivery systems necessitates the covalent conjugation of therapeutic molecules to natural polymers through carefully designed hydrolysable linkages, including amide, hydrazone, and ester bonds, to achieve controlled release kinetics [38]. In the present study, we employed Steglich esterification to conjugate DEX with HA, a well-established method for forming ester bonds between carboxyl and hydroxyl groups through carbodiimide activation in the presence of DMAP as a catalyst. This synthetic approach is particularly advantageous for modifying labile compounds containing multiple reactive sites, as it proceeds under mild conditions with high efficiency [39]. Using EDC and DMAP as activators of HA’s carboxyl groups, we successfully synthesized HA-DEX conjugates through formation of an ester linkage between the C21 hydroxyl group of DEX and the carboxyl group of HA’s glucuronic acid units (Figure 2a).

The reaction mechanism (Figure 2b) involves several key steps: Initially, EDC reacts with the carboxyl group of HA to form an O-acylisourea intermediate, which exhibits reactivity comparable to that of carboxylic acid anhydrides. While EDC primarily serves as a condensing agent for amide bond formation, this intermediate can undergo slow acyl migration to produce N-acylurea byproducts. DMAP, functioning as a highly effective nucleophilic catalyst, reacts preferentially with the O-acylisourea intermediate to generate a more stable active ester species (N-acyl-4-dimethylaminopyridinium intermediate). This activated intermediate cannot form intramolecular byproducts and instead reacts efficiently with the hydroxyl group of DEX to yield the desired ester bond. The DMAP-mediated acyl transfer mechanism thus ensures high reaction specificity while minimizing side product formation.

**Figure 3:**
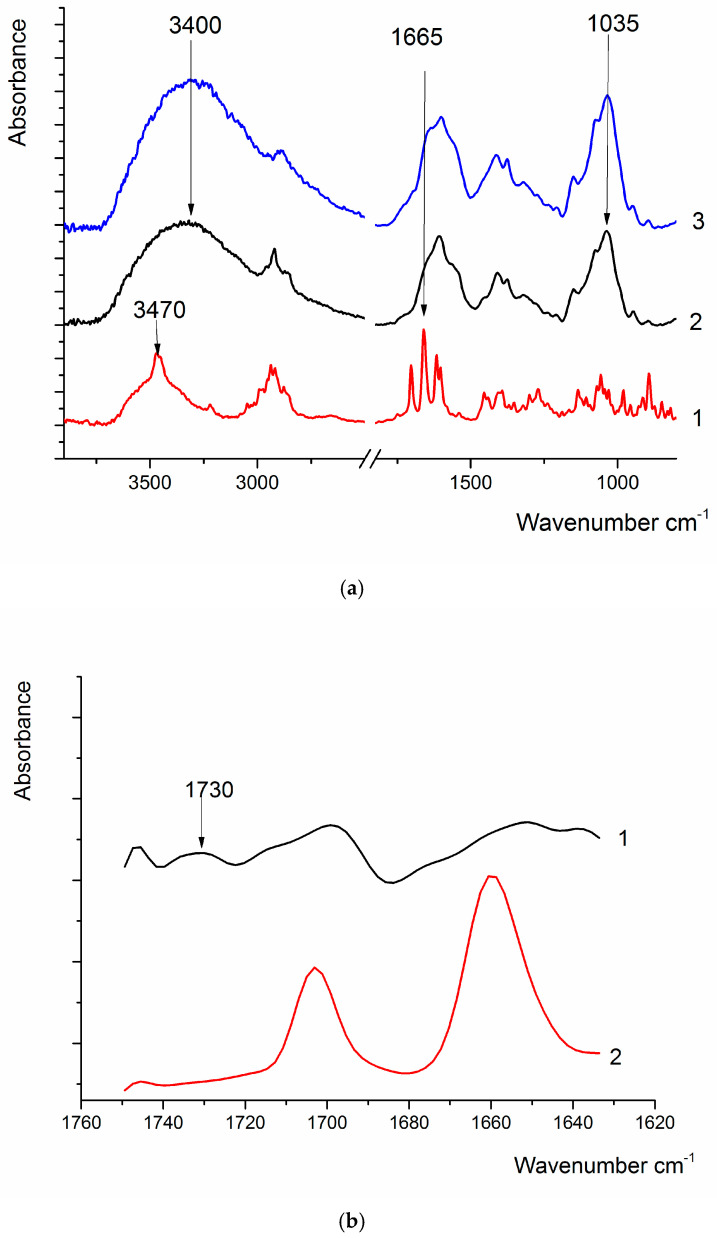
(**a**) FTIR spectra of DEX (1), HA (2), and HA-DEX (3); (**b**) FTIR spectrum of DEX (1) and the difference spectrum of HA-DEX and HA (2), obtained via spectral deconvolution.

Characterization of the HA-DEX conjugates was performed using Fourier transform infrared (FTIR) spectroscopy to confirm ester bond formation (Figure 3). Figure 3a presents the comparative FTIR spectra of pure DEX, pure HA, and the HA-DEX conjugate. While the ester carbonyl stretch at 1730 cm^−1^ was observable in the HA-DEX spectrum, its intensity was relatively weak due to the low degree of DEX substitution. To unequivocally demonstrate the presence of this ester linkage, we generated a difference spectrum by digitally subtracting the HA spectrum from the HA-DEX conjugate spectrum; spectral deconvolution was performed by setting the curve to a Lorentzian shape with a signal-to-noise ratio of 0.5. The resulting bands’ half-width was maintained within the range of 15–20 cm^−1^ (Figure 3b). This differential analysis revealed two key features: first, the appearance of a distinct absorption band at 1730 cm^−1^ corresponding to the newly formed ester linkage between HA and DEX (absent in both parent compounds), and second, the presence of DEX’s characteristic keto group absorption at 1760 cm^−1^, confirming successful incorporation of the steroid moiety into the conjugate.

**Figure 4:**
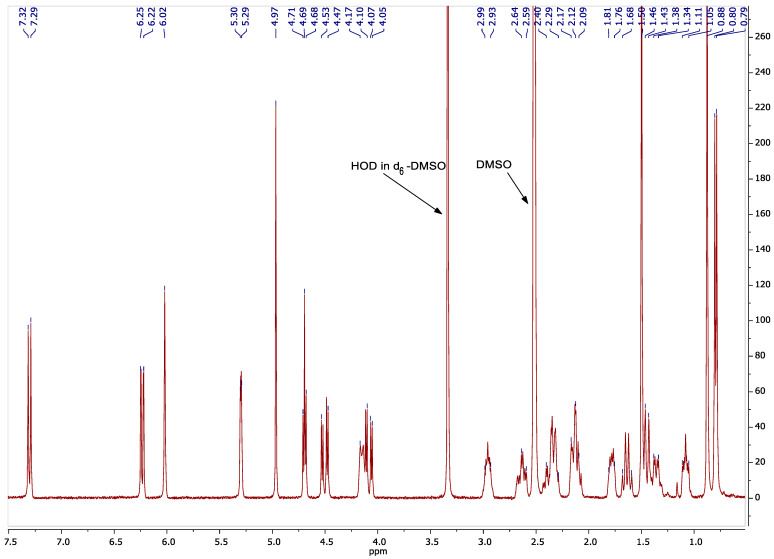
^1^H NMR (400 MHz, dimethyl sulfoxide -d6) spectrum of DEX, δ 7.31 (d, 1H), 6.25-6.22 (dd, 1H), 6.02 (s, 1H), 5.30-5.29 (dd, 1H), 4.97 (s, 1H), 4.71-4.68 (t, 1H), 4.53-4.48 (dd, 1H), 4.17-4.10 (m, 1H), 4.06 (d, 1H), 2.99-2.93 (m, 1H), 2.64-2.59 (m, 1H), 2.52-2.51 (t, 1H), 2.4-2.30 (m, 1H), 2.17-2.12 (m, 1H), 2.09 (d, 1H), 1.81-1.76 (m, 1H), 1.68-1.59 (m, 1H), 1.50 (s, 3H), 1.46-1.44 (m, 1H), 1.35-1.38 (dd, 1H), 1.11-1.05 (m, 1H), 0.88 (s, 3H), and 0.80 (d, 3H).

**Figure 5:**
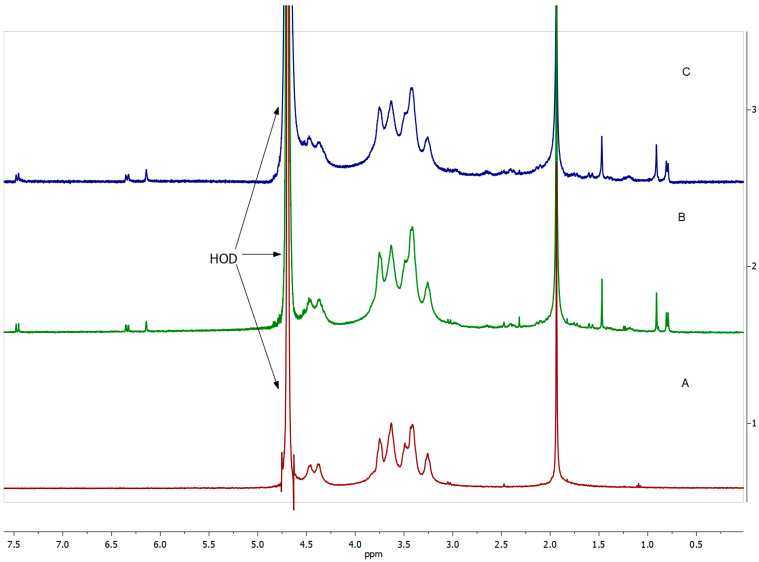
^1^H NMR spectra (400 MHz, D_2_O) of (A) HA, (B) HA-DEX-5, and (C) HA-DEX-10.

Further structural characterization was achieved through ^1^H nuclear magnetic resonance (NMR) spectroscopy (400 MHz, D_2_O), which enabled the quantitative determination of the degree of DEX substitution (DS). The ^1^H NMR spectrum of DEX displayed characteristic proton resonances (Figure 4). In the HA-DEX conjugates (Figure 5), we observed all characteristic HA proton signals, including the methyl group protons (-CH_3_) from N-acetyl-D-glucosamine at 1.94 ppm, the complex multiplet between 3.2 and 3.8 ppm corresponding to H-2 through H-6 protons of both sugar units, and the anomeric proton signals at 4.38 and 4.48 ppm. Additionally, multiple DEX proton resonances appeared between 0.7 and 1.8 ppm. Using the integrated intensity of the acetamide methyl protons at 1.94 ppm (set to 1.5) as a reference, we calculated DS according to

$$DS=\frac{{6I(DEX)}_{0.7-1.8}}{14I(H-2-H-6)},$$

where I(DEX)0.7–1.8 represents the integrated intensity of DEX protons in the 0.7–1.8 ppm region (corresponding to 14 protons) and I(H-2-H-6) is the integrated intensity of HA’s ring protons. This analysis revealed DS values ranging from 2% to 5% across different conjugate batches (Table 1). These calculations indicate a maximum theoretical DS of 50%, as only the glucuronic acid residues (constituting half of HA’s disaccharide repeating units) contain carboxyl groups available for conjugation.

The aqueous phase behavior and surface characteristics of the HA-DEX conjugates were investigated to evaluate their potential as nanocarriers (Table 2). When dispersed in aqueous media, the amphiphilic conjugates spontaneously self-organized into nanoparticles exhibiting hydrodynamic diameters between 240 and 600 nm as quantified by dynamic light scattering analysis. The hydrodynamic diameters remained consistent within the margin of error in water and buffer solutions at pH levels of 7.4 and 5.2. This suggests that the self-assembled polymer particles are stable in various aqueous environments. A distinct correlation was observed between increasing particle size and higher DS, indicative of enhanced hydrophobic interactions mediated by the incorporated DEX moieties. Electrophoretic mobility measurements demonstrated that all formulations possessed negatively charged surfaces, with ζ-potential values ranging from −21 to −25 mV. Interestingly, the surface charge density exhibited a clear dependence on DS, with greater DEX incorporation resulting in progressively higher negative ζ-potentials.

**Figure 6:**
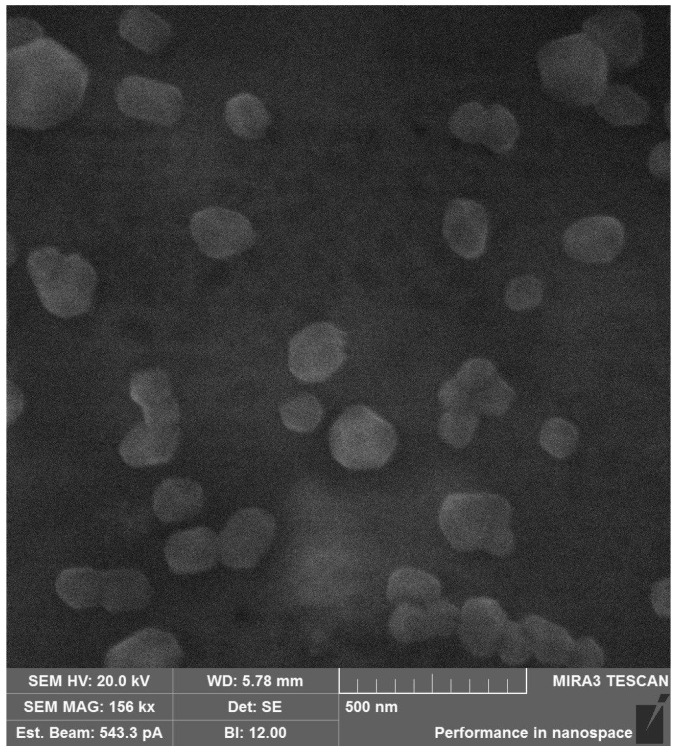
SEM image of the HA-DEX-5 particles.

Morphological examination by SEM (Figure 6) revealed that the HA-DEX-5 conjugate formed discrete, spherical nanoparticles with narrow size distribution and smooth surface topography. The combined physicochemical characterization confirms the successful engineering of stable, self-assembled nanostructures through the rational design of HA-DEX conjugates, where the ester linkages provide controlled drug release capability while maintaining the advantageous properties of the HA backbone for sustained delivery applications.

### 2.2. In Vitro DEX Release Kinetics from HA-DEX Conjugates

**Figure 7:**
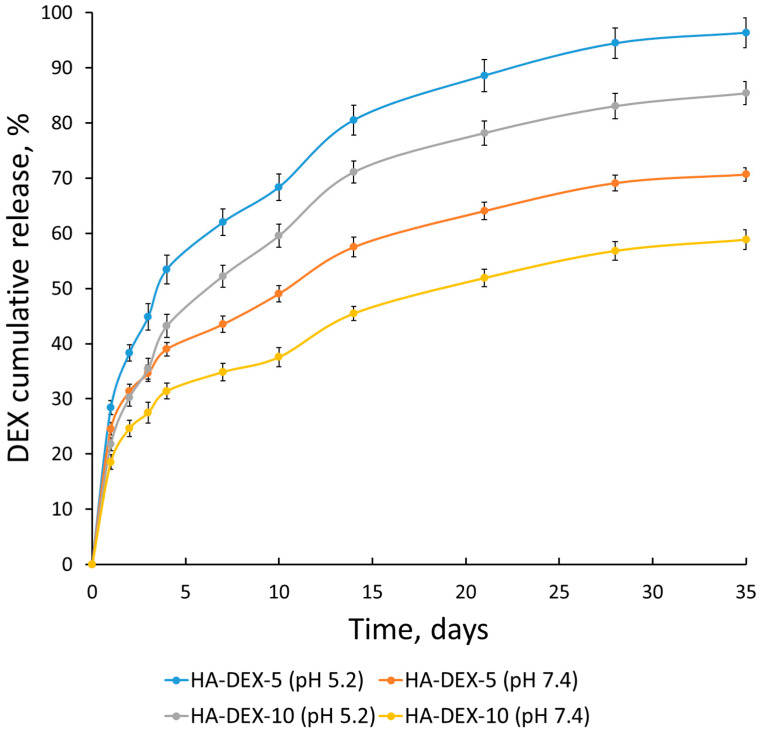
Release kinetics of DEX from the HA-DEX particles in PB with pH 5.2 and PBS with pH 7.4 at 37 °C; *n* = 3, error bars represent standard deviation.

The HA-DEX conjugates were designed with ester linkages between the polymer carrier and drug molecules to enable sustained release through controlled hydrolytic cleavage. To evaluate the release kinetics under physiologically relevant conditions, we conducted in vitro studies using two buffer systems: PBS pH 7.4 simulating normal physiological parameters (ionic strength, osmolarity, and pH of bloodstream), and PB pH 5.2 mimicking the acidic microenvironment characteristic of inflammatory sites. This dual-pH approach allowed for the systematic investigation of both the hydrolysis rate and pH-sensitivity of the ester bonds in our conjugates. Figure 7 presents the cumulative DEX release profiles from HA-DEX-5 and HA-DEX-10 formulations under these conditions at physiological temperature (37 °C). The results demonstrate significant pH-dependent release behavior, with approximately 80–90% of DEX released over one month in acidic conditions (PB, pH 5.2) compared to 50–60% release in neutral buffer (PBS, pH 7.4). This differential release profile, combined with the sustained delivery over an extended period, highlights the potential of these HA-DEX conjugates as targeted anti-inflammatory therapeutics capable of responding to the acidic microenvironment characteristic of inflamed tissues while maintaining controlled release under normal physiological conditions. The pH-modulated release kinetics suggest these systems may preferentially deliver higher drug concentrations to sites of inflammation while minimizing systemic exposure, representing a promising approach for localized anti-inflammatory therapy.

Assuming a complex drug release mechanism involving both the hydrolysis of chemically bound DEX and the release of hydrolyzed DEX physically entrapped within the hydrophobic core of the HA-DEX conjugate, we analyzed the release kinetics using the Korsmeyer–Peppas model [40,41]

$${Q=K}_{KP}t^{n},$$

where *Q* represents the cumulative percentage of DEX released, *K_KP_* is the release rate constant, *n* is the release exponent, and *t* denotes the release time in hours [41].

**Figure 8:**
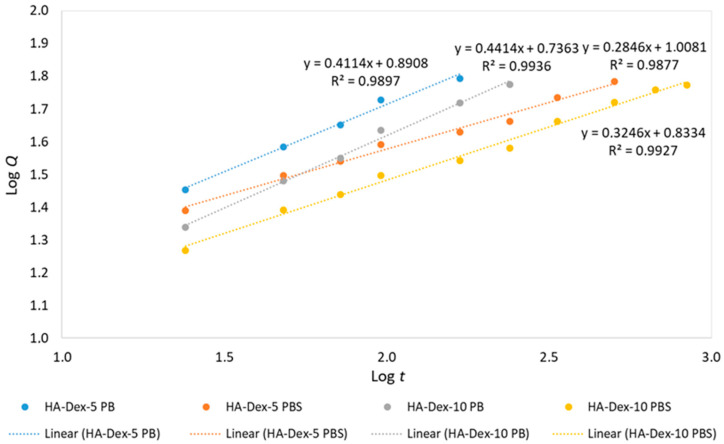
Linearized release kinetics of DEX from HA-DEX conjugates.

Linearized DEX release kinetics curve fitting was performed in Log *Q*-Log *t* coordinates within the *Q* range of 15% to 60%, as recommended in [41]. The fitted parameters are summarized in Table 3 and presented in Figure 8. The fitting results obtained using the Korsmeyer–Peppas kinetic model formally indicate that the predominant mechanism of DEX release from the polymer conjugate is Fickian diffusion (n ≤ 0.45). However, it should be noted that in the case of covalently bound conjugates, the drug release rate constitutes a complex process. This process is predominantly associated with the sequential hydrolysis of ester bonds in the HA-DEX conjugate and a combination of transfer mechanisms, including the dissolution of DEX within the hydrophobic glucocorticoid core followed by diffusion through the hydrophilic polysaccharide shell.

### 2.3. Anti-Inflammatory Activity of HA-DEX Conjugates

#### 2.3.1. Evaluation of the Effect of HA-DEX Conjugates on THP-1 Cell Viability

The cytotoxic potential of HA-DEX conjugates was systematically evaluated using the THP-1 monocytic cell line, with comparative assessment of free HA and free DEX at equivalent concentrations (1 μg/mL and 10 μg/mL DEX). Comprehensive viability analysis through multiparametric flow cytometry revealed no significant alterations in cell viability across all experimental conditions. Following 24 h incubation periods, quantitative assessment demonstrated maintenance of baseline viability levels, with preserved percentages of viable cells (YO-PRO-1–PI– phenotype) in both unstimulated cultures and TNFα-activated conditions (2 ng/mL final concentration) (Table 4). Detailed examination of apoptotic progression showed consistent values for early apoptotic populations (YO-PRO-1+PI– phenotype, Table 5) and late apoptotic/necrotic fractions (YO-PRO-1+PI+ phenotype, Table 6) across all treatment groups at a 1 μg/mL DEX concentration.

This non-cytotoxic profile was further confirmed at the higher test concentration (10 μg/mL DEX equivalent), where analogous results were obtained for viable cell populations (Table 7), early apoptotic indices (Table 8), and terminal apoptotic/necrotic fractions (Table 9). The consistent absence of statistically significant differences in viability parameters between conjugate-treated groups and controls (free HA, free DEX, and untreated cells) under both resting and TNFα-stimulated conditions demonstrates the favorable biocompatibility profile of the HA-DEX delivery system across therapeutically relevant concentration ranges.

#### 2.3.2. Evaluation of the Effect of HA-DEX Conjugates on the Blockade of THP-1 Cell Activation by TNFα

The immunomodulatory effects of HA-DEX conjugates were evaluated by assessing their capacity to inhibit the TNFα-induced activation of THP-1 cells, as measured by CD54 (ICAM-1) surface expression. TNFα stimulation at 2 ng/mL significantly upregulated CD54 expression (median fluorescence intensity [MFI] increasing from 0.609 [0.551; 0.679] to 4.452 [4.164; 5.232], *p* < 0.001) (Table 10). Under basal conditions, neither HA-DEX conjugates, free HA, nor free DEX (1 μg/mL) altered CD54 expression. However, in TNFα-activated cultures, free DEX significantly suppressed CD54 levels (MFI reduction from 4.452 [4.164; 5.232] to 2.122 [1.357; 2.330], *p* < 0.001), while free HA showed no effect. Notably, both HA-DEX-5 and HA-DEX-10 demonstrated potent anti-inflammatory activity, reducing CD54 expression to 1.694 [1.270; 2.353] and 1.791 [1.300; 2.125] MFI, respectively (*p* < 0.001 vs. TNFα control), with efficacy comparable to free DEX (*p* = 0.757 for HA-DEX-5; *p* = 0.791 for HA-DEX-10) and no significant difference between conjugates (*p* = 0.427).

At higher concentrations (10 μg/mL DEX equivalent), free DEX paradoxically increased basal CD54 expression (3.203 [2.307; 3.748] vs. 0.609 [0.551; 0.679], *p* = 0.001), suggesting concentration-dependent immunostimulatory effects (Table 11). Under TNFα stimulation at this concentration, only HA-DEX conjugates maintained significant anti-inflammatory activity, reducing CD54 to 2.514 [2.057; 2.597] (HA-DEX-5) and 2.237 [1.428; 2.485] MFI (HA-DEX-10) (both *p* < 0.001 vs. TNFα control), while free DEX lost efficacy (MFI 5.129 [3.983; 6.054], *p* = 0.894). Importantly, HA-DEX conjugates also attenuated the aberrant CD54 elevation induced by high-dose free DEX (*p* < 0.001 for both conjugates vs. free DEX control), with no significant difference between HA-DEX-5 and HA-DEX-10 (*p* = 0.402). These results demonstrate that HA conjugation preserves DEX’s anti-inflammatory effects while preventing the pro-inflammatory responses observed with high concentrations of the free drug.

## 3. Materials and Methods

### 3.1. Materials and Reagents

Sodium hyaluronate with a viscosity average molecular weight of 180,000 was used in this study [42]. Dexamethasone, 1-ethyl-3-(3-dimethylaminopropyl)carbodiimide hydrochloride, 4-dimethylaminopyridine, PBS tablets, dimethyl sulfoxide, and deuterium oxide (D_2_O, 99.9 atom % D) were purchased from Sigma-Aldrich Co. (St. Louis, MO, USA). All other chemicals and solvents were obtained from commercial suppliers and used without further purification.

### 3.2. Synthesis of HA-DEX Conjugates

The conjugation procedure was initiated by dissolving HA (0.1 g, 3.082% nitrogen content, 0.44 mmol of sugar units) in 20 mL of deionized water. Carboxyl group activation was achieved by adding EDC and DMAP to the HA solution, followed by 30 min of stirring at room temperature. Separately, DEX was dissolved in 3 mL of dimethyl sulfoxide and subsequently added to the activated HA solution. The reaction mixture was maintained at 20 °C with continuous stirring for 72 h (specific molar ratios of HA:EDC:DMAP:DEX are detailed in Table 1).

The crude product was isolated by acetone precipitation, followed by two washing steps with fresh acetone and filtration. The purified conjugate was obtained by redissolving the precipitate in distilled water, dialyzing (12,000–14,000 MWCO membrane) against distilled water for 3 days to remove unreacted reagents, and finally lyophilizing using a freeze-dryer (Fanbolun Ltd., Guangzhou, China).

### 3.3. Characterization of HA-DEX Conjugates

FTIR spectroscopy was performed in attenuated total reflection (ATR) mode using a Vertex-70 spectrometer (Bruker, Billerica, MA, USA) equipped with a ZnSe crystal accessory (PIKE Technologies, Fitchburg, WI, USA). UV-Vis absorption spectra were acquired using a UV-1700 PharmaSpec spectrophotometer (Shimadzu, Kyoto, Japan).

^1^H NMR spectra were recorded on a Bruker Avance 400 MHz spectrometer (Bruker, Billerica, MA, USA). For analysis, 5 mg of HA-DEX conjugates were dissolved in D_2_O.

Particle size distribution (hydrodynamic diameter, D_h_) and ζ-potential were determined by dynamic light scattering and electrophoretic light scattering, respectively, using a Photocor Compact-Z system (Photocor, Moscow, Russia) equipped with a 659.7 nm He-Ne laser (25 mW output). Measurements were conducted at 20 °C with a detection angle of 90°.

Morphological characterization was performed by scanning electron microscopy (SEM) using a Tescan Mira 3 microscope (Tescan, Brno, Czech Republic) operated in secondary electron (SE) mode at a 20 kV accelerating voltage and 543.3 pA beam current. Samples were prepared by deposition onto carbon tape followed by 24 h vacuum drying, with a working distance of 6 mm maintained during imaging.

Quantification of DEX loading was achieved by UV-Vis spectrophotometry at 242 nm (UV-1700 PharmaSpec, Shimadzu). Calibration curves were established using standard DEX solutions, and sample concentrations were adjusted to 1 mg HA-DEX per 20 mL deionized water for measurement. Drug loading was expressed as μg DEX per mg conjugate.

### 3.4. In Vitro DEX Release Kinetics

The release profile of DEX from HA-DEX conjugates was evaluated under physiologically relevant conditions using two different buffer systems: phosphate buffer (PB, pH 5.2) and PBS pH 7.4. For each release study, 4 mg of HA-DEX conjugate was dispersed in 4 mL of the respective buffer medium and incubated at 37 °C for 35 days under constant agitation.

At predetermined time points, aliquots were collected and subjected to ultracentrifugation at 4500 rpm using Vivaspin^®^ Turbo4 5000 molecular weight cut-off centrifugal concentrators (JetBio-Filtration, Guangzhou, China). Following each sampling, an equivalent volume of fresh buffer was added to maintain sink conditions throughout the study period.

The concentration of released DEX in the supernatant was quantified spectrophotometrically at 242 nm using a UV-1700 PharmaSpec spectrophotometer (Shimadzu, Kyoto, Japan). Quantification was achieved by comparison with a pre-established calibration curve of DEX standards in the respective buffer systems. All measurements were performed in triplicate to ensure reproducibility.

### 3.5. Anti-Inflammatory Properties of HA-DEX Conjugates

#### 3.5.1. THP-1 Cell Culture and Activation

The human acute monocytic leukemia THP-1 cell line was obtained from the Vertebrate Cell Culture Collection of the Institute of Cytology, Russian Academy of Sciences. Cells were maintained in RPMI-1640 medium (Biolot, St. Petersburg, Russia) supplemented with 10% heat-inactivated fetal bovine serum (Life Technologies, Carlsbad, CA, USA), 50 μg/mL gentamicin (Biolot, St. Petersburg, Russia), and 2 mM L-glutamine (Biolot, St. Petersburg, Russia). Cultures were grown in tissue culture flasks (Sarstedt, Nümbrecht, Germany) at 37 °C in a humidified 5% CO_2_ atmosphere, with subculturing performed every 2–3 days to maintain logarithmic growth.

For activation experiments, THP-1 cells were stimulated with recombinant human tumor necrosis factor-α (TNFα; BioLegend, San Diego, CA, USA) at a final concentration of 2 ng/mL, while unstimulated cells served as negative controls.

For substance testing, cell suspensions (200 μL containing 1 × 10^5^ cells) were aliquoted into 96-well plates (Sarstedt, Nümbrecht, Germany) and incubated with test compounds for 24 h. Following incubation, the cells were transferred to 75 × 12 mm flow cytometry tubes (Sarstedt, Nümbrecht, Germany), washed with 4 mL sterile PBS, and centrifuged to obtain cell pellets.

The pellets were resuspended in 100 μL fresh PBS and stained with mouse anti-human CD54 monoclonal antibodies (Beckman Coulter, Brea, CA, USA) for 15 min at room temperature in the dark, following established protocols [13]. After antibody staining, the cells were washed with PBS and counterstained with 4′,6-diamidino-2-phenylindole (DAPI; 1 μg/mL final concentration, BioLegend, San Diego, CA, USA) for live/dead cell discrimination.

Flow cytometry analysis was performed with the acquisition of at least 10,000 events per sample to ensure statistical significance. All experiments were conducted in biological triplicates to verify reproducibility.

#### 3.5.2. Assessment of Cell Viability Using DNA-Binding Dyes

The assessment of cell viability was performed using the fluorescent DNA-binding dyes YO-PRO-1 iodide (629.3 Da, Thermo Fisher Scientific, Waltham, MA, USA) and propidium iodide (PI; 668.4 Da, Sigma-Aldrich, St. Louis, MO, USA) through a dual-staining approach [43]. Both dyes exhibit stoichiometric binding to cellular nucleic acids, accumulating in the cytoplasm through RNA interaction and in the nucleus via DNA binding, resulting in characteristic fluorescence shifts upon 488 nm excitation. YO-PRO-1 demonstrates peak emission at 509 nm (green spectrum), while PI emits at 617 nm (red spectrum). Their differential cellular uptake mechanisms provide distinct apoptotic stage detection: YO-PRO-1 enters viable cells exclusively through activated P2RX7 purinergic ion channels [44,45], which remain inactive in healthy cells but become operational during early apoptosis coinciding with membrane phospholipid asymmetry disruption. In contrast, PI lacks specific membrane transporters and only penetrates cells with compromised membrane integrity, typically occurring during late apoptosis or necrosis. This differential permeability allows for the precise staging of cell death processes—viable cells remain unstained by both dyes, early apoptotic cells show YO-PRO-1 positivity alone, and late apoptotic cells exhibit dual staining with both fluorophores. The combination thus enables discrimination between viable, early apoptotic, late apoptotic, and necrotic cell populations based on their distinct staining patterns.

The cell staining and flow cytometry analysis were performed using standardized protocols in 12 × 75 mm cytometry tubes (Beckman Coulter, Brea, CA, USA). Cell suspensions (100 μL containing 2–3 × 10^6^ cells/mL) were treated with 5 μL of freshly prepared 20× YO-PRO-1 working solution (Thermo Fisher Scientific, Waltham, MA, USA), yielding a final concentration of 250 nM. The working solution was prepared immediately before use by diluting 10 μL of stock solution (100 μM in dimethyl sulfoxide, aliquoted and stored at −20 °C) with 190 μL of PBS. Following 15 min of incubation at room temperature in the dark, 10 μL of propidium iodide solution (Sigma-Aldrich, St. Louis, MO, USA) was added to achieve a final PI concentration of 1 μg/mL. After adding 200 μL of PBS to each sample, cellular analysis was conducted on a Navios™ flow cytometer (Beckman Coulter, Brea, CA, USA) with an acquisition of at least 20,000 single-cell events per sample. Single cells were discriminated from aggregates using multiparametric gating strategies based on forward scatter (FS) and side scatter (SS) parameters, specifically evaluating peak versus integral signal intensity correlations and time-of-flight versus signal intensity relationships. All data analysis was performed using Kaluza™ (version 2.1) analysis software (Beckman Coulter, Brea, CA, USA), enabling the precise quantification of cell populations based on their differential staining characteristics.

## 4. Conclusions

This study successfully developed and characterized novel HA-DEX conjugates synthesized via direct Steglich esterification without linker molecules. The one-step carbodiimide-mediated reaction between HA carboxyl groups and the C21 hydroxyl of DEX yielded pH-responsive nanoparticles with well-defined physicochemical properties. The conjugates self-assembled into spherical nanoparticles (240–600 nm) exhibiting negative ζ-potential (−20 to −25 mV), which confers colloidal stability in biological fluids. Comprehensive characterization confirmed ester bond formation and controlled DEX substitution degrees (2–5%).

Key functional advantages include the following:

i. Sustained pH-dependent drug release, with 80–90% DEX released at inflammatory pH (5.2) versus 50–60% at physiological pH (7.4) over 35 days.
ii. Significant anti-inflammatory activity in TNFα-induced inflammation model, effectively suppressing CD54 expression in THP-1 monocytes.
iii. Absence of cytotoxicity across tested formulations, confirming biocompatibility.

The combination of HA’s CD44-targeting capability, EPR-driven passive accumulation, and pH-responsive release kinetics positions these conjugates as promising platforms for localized glucocorticoid delivery. Their stability in physiological environments (blood, synovial fluid, vitreous humor) and organ-specific release profiles support potential applications in intra-articular, intraocular, and intratympanic therapies for inflammatory conditions.

## Figures and Tables

**Table 1:** Synthesis conditions and characterization of HA-DEX conjugates.

Sample	Molar Ratio of Reagents				DS (%) by NMR	DEX-Content (μg/mg)
	HA	DMAP	EDC	DEX		
HA-DEX-3	1	0.2	4	0.03	2.1	-
HA-DEX-5	1	0.2	4	0.05	3.1	31
HA-DEX-10	1	0.2	4	0.1	5.0	53

**Table 2:** Characteristics of HA-DEX particles in aqueous solutions (mean ± standard deviation, *n* = 3).

Sample	D_h_ (nm)	ζ-Potential (mV)
HA-Dex-3	244 ± 86	−20.9 ± 0.9
HA-Dex-5	330 ± 78	−21.3 ± 0.7
	259 ± 50 *	
	348 ± 92 **	
HA-Dex-10	604 ± 166	−25.6 ± 0.8
	645 ± 54 *	
	650 ± 80 **	

* D_h_ in phosphate-buffered saline (PBS) at pH 7.4; ** D_h_ in phosphate buffer (PB) at pH 5.2.

**Table 3:** Fitting parameters of DEX release kinetics based on the Korsmeyer–Peppas model.

Sample	K_KP_	n	R^2^
HA-DEX-5 (PB pH 5.2)	7.8	0.411	0.9897
HA-DEX-5 (PBS pH 7.4)	10.2	0.285	0.9877
HA-DEX-10 (PB pH 5.2)	5.4	0.441	0.9936
HA-DEX-10 (PBS pH 7.4)	6.8	0.325	0.9927

**Table 4:** Relative live cell content of the THP-1 cell line with YO-PRO-1–PI– phenotype after 24 h incubation in the presence of HA-DEX conjugates. Results (*n* ≥ 9) are shown as median and interquartile range, Me (Q25; Q75).

Sample	No TNFα	2 ng/mL TNFα
Negative control	95.82 (95.50; 96.57)	95.87 (95.31; 96.14)
DEX	96.23 (95.92; 96.35)	95.47 (95.39; 95.64)
HA	96.20 (96.18; 96.35)	95.95 (95.42; 96.03)
HA-DEX-5	96.34 (96.20; 96.43)	95.31 (95.17; 95.62)
HA-DEX-10	96.38 (95.90; 96.62)	95.75 (95.46; 95.82)

**Table 5:** Relative content of THP-1 cell line cells in early stages of apoptosis (YO-PRO-1+PI– phenotype) after 24 h incubation in the presence of HA-DEX conjugates. Results (*n* ≥ 9) are shown as median and interquartile range, Me (Q25; Q75).

Sample	No TNFα	2 ng/mL TNFα
Negative control	1.97 (1.56; 2.51)	2.43 (1.78; 2.93)
DEX	1.41 (1.15; 2.10) *	2.63 (1.74; 3.34)
HA	1.94 (1.64; 2.08)	2.66 (1.98; 2.86)
HA-DEX-5	1.96 (1.84; 1.97)	3.00 (2.63; 3.67)
HA-DEX-10	1.90 (1.27; 2.57)	3.01 (2.38; 3.18)

*—differences with negative control (THP-1 without addition of HA-DEX) are significant at *p* < 0.05 according to the nonparametric Mann–Whitney test.

**Table 6:** Relative content of THP-1 cell line cells in late stages of apoptosis and/or necrosis (YO-PRO-1+PI+ phenotype) after 24 h incubation in the presence of HA-DEX conjugates. Results (*n* ≥ 9) are shown as median and interquartile range, Me (Q25; Q75).

Sample	No TNFα	2 ng/mL TNFα
Negative control	1.89 (1.71; 2.72)	1.80 (1.48; 2.62)
DEX	2.32 (1.94; 2.74)	1.94 (1.25; 2.04)
HA	1.78 (1.73; 2.17)	1.80 (1.07; 2.01)
HA-DEX-5	1.69 (1.61; 1.84)	1.31 (1.22; 1.75)
HA-DEX-10	1.72 (1.50; 2.05)	1.07 (0.96; 2.08)

**Table 7:** Relative live cell content of the THP-1 cell line with YO-PRO-1–PI– phenotype after 24 h incubation in the presence of HA-DEX conjugates. Results (*n* ≥ 9) are shown as median and interquartile range, Me (Q25; Q75).

Sample	No TNFα	2 ng/mL TNFα
Negative control	95.82 (95.50; 96.57)	95.87 (95.31; 96.14)
DEX	95.88 (95.82; 96.19)	95.82 (95.51; 95.92)
HA	96.38 (95.73; 96.46)	96.03 (95.80; 96.11)
HA-DEX-5	96.18 (95.79; 96.62)	95.56 (95.42; 95.92)
HA-DEX-10	96.16 (96.01; 96.56)	95.98 (95.89; 96.01)

**Table 8:** Relative content of THP-1 cell line cells in early stages of apoptosis (YO-PRO-1+PI– phenotype) after 24 h incubation in the presence of HA-DEX conjugates. Results (*n* ≥ 9) are shown as median and interquartile range, Me (Q25; Q75).

Sample	No TNFα	2 ng/mL TNFα
Negative control	1.97 (1.56; 2.51)	2.43 (1.78; 2.93)
DEX	2.68 (0.77; 2.80)	2.97 (1.32; 3.19)
HA	1.93 (1.54; 2.24)	2.18 (2.02; 2.54)
HA-DEX-5	2.47 (1.79; 3.15)	3.05 (2.76; 3.47) *
HA-DEX-10	1.90 (1.67; 2.71)	3.11 (2.42; 3.31)

*—differences with negative control are significant at *p* < 0.05 according to the Mann–Whitney test.

**Table 9:** Relative content of THP-1 cell line cells in late stages of apoptosis and/or necrosis (YO-PRO-1+PI+ phenotype) after 24 h incubation in the presence of HA-DEX conjugates. Results (*n* ≥ 9) are shown as median and interquartile range, Me (Q25; Q75).

Sample	No TNFα	2 ng/mL TNFα
Negative control	1.89 (1.71; 2.72)	1.80 (1.48; 2.62)
DEX	1.45 (1.38; 2.84)	1.80 (1.26; 2.71)
HA	1.98 (1.60; 2.16)	1.79 (1.35; 2.05)
HA-DEX-5	1.36 (0.74; 1.83) *	1.21 (1.06; 1.60)
HA-DEX-10	1.72 (1.23; 2.14)	1.11 (0.67; 1.70) *

*—differences with negative control are significant at *p* < 0.05 according to the Mann–Whitney test.

**Table 10:** Effect of HA-DEX conjugates (1 μg/mL DEX) on the activation level of the THP-1 cell line. Results (*n* = 9) are presented as conditional CD54 fluorescence units (MFI units) and summarized as median and interquartile range, Me (Q25; Q75).

Sample	No TNFα	2 ng/mL TNFα
Negative control	0.609 (0.551; 0.679)	4.452 (4.164; 5.232)
DEX	0.583 (0.567; 0.593)	2.122 (1.357; 2.330) ***
HA	0.561 (0.444; 0.583)	5.573 (3.430; 6.332)
HA-DEX-5	0.549 (0.520; 0.592)	1.694 (1.270; 2.353) ***
HA-DEX-10	0.566 (0.528; 0.567)	1.791 (1.300; 2.125) ***

***—differences with negative control are significant at *p* < 0.001 according to the Mann–Whitney test.

**Table 11:** Effect of HA-DEX conjugates (10 μg/mL DEX) on the activation level of the THP-1 cell line. Results (*n* = 9) are presented as conditional CD54 fluorescence units (MFI units) and summarized as median and interquartile range, Me (Q25; Q75).

Sample	No TNFα	2 ng/mL TNFα
Negative control	0.609 (0.551; 0.679)	4.452 (4.164; 5.232)
DEX	3.203 (2.307; 3.748)	5.129 (3.983; 6.054)
HA	0.550 (0.437; 0.590)	4.715 (3.981; 6.346)
HA-DEX-5	0.772 (0.650; 0.864)	2.514 (2.057; 2.597)
HA-DEX-10	0.606 (0.594; 0.641)	2.237 (1.428; 2.485)

## Data Availability

The original contributions presented in this study are included in the article. Further inquiries can be directed to the corresponding authors.
